# Induction of Tier 1 HIV Neutralizing Antibodies by Envelope Trimers Incorporated into a Replication Competent Vesicular Stomatitis Virus Vector

**DOI:** 10.3390/v11020159

**Published:** 2019-02-15

**Authors:** C. Anika Bresk, Tamara Hofer, Sarah Wilmschen, Marina Krismer, Anja Beierfuß, Grégory Effantin, Winfried Weissenhorn, Michael J. Hogan, Andrea P. O. Jordan, Rebecca S. Gelman, David C. Montefiori, Hua-Xin Liao, Joern E. Schmitz, Barton F. Haynes, Dorothee von Laer, Janine Kimpel

**Affiliations:** 1 Division of Virology, Medical University of Innsbruck, 6020 Innsbruck, Austria; ANIKA.BRESK@I-MED.AC.AT (C.A.B.); tamara.hofer@i-med.ac.at (T.H.); sarah.wilmschen@i-med.ac.at (S.W.); marina.krismer@gmail.com (M.K.); Dorothee.von-Laer@i-med.ac.at (D.v.L.); 2 Christian Doppler Laboratory for Viral Immunotherapy of Cancer, Medical University of Innsbruck, 6020 Innsbruck, Austria; 3 Central Laboratory Animal Facility, Medical University of Innsbruck, 6020 Innsbruck, Austria; Anja.Beierfuss@i-med.ac.at; 4 Institut de Biologie Structurale (IBS), CNRS, CEA, Université Grenoble Alpes, 38044 Grenoble, France; gregory.effantin@ibs.fr (G.E.); winfried.weissenhorn@ibs.fr (W.W.); 5 Division of Hematology and Oncology, Perelman School of Medicine, University of Pennsylvania, Philadelphia, PA 19104, USA; hoganm@email.chop.edu (M.J.H.); andreado@pennmedicine.upenn.edu (A.P.O.J.); 6 Dana-Farber Cancer Institute, Harvard Medical School and Harvard School of Public Health, Boston, MA 02215, USA; gelman@jimmy.harvard.edu; 7 Duke Human Vaccine Institute, Duke University School of Medicine, Durham, NC 27710, USA; david.montefiori@duke.edu (D.C.M.); l.liao@duke.edu (H.-X.L.); barton.haynes@duke.edu (B.F.H.); 8 Center for Virology and Vaccine Research, Beth Israel Deaconess Medical Center, Harvard Medical School, Boston, MA 02115, USA; joern.e.schmitz@gmail.com

**Keywords:** HIV vaccine, vesicular stomatitis virus, VSV-GP viral vaccine vector, 1086.C HIV-1 Env, broadly neutralizing antibodies

## Abstract

A chimeric vesicular stomatitis virus with the glycoprotein of the lymphocytic choriomeningitis virus, VSV-GP, is a potent viral vaccine vector that overcomes several of the limitations of wild-type VSV. Here, we evaluated the potential of VSV-GP as an HIV vaccine vector. We introduced genes for different variants of the HIV-1 envelope protein Env, i.e., secreted or membrane-anchored, intact or mutated furin cleavage site or different C-termini, into the genome of VSV-GP. We found that the addition of the Env antigen did not attenuate VSV-GP replication. All HIV-1 Env variants were expressed in VSV-GP infected cells and some were incorporated very efficiently into VSV-GP particles. Crucial epitopes for binding of broadly neutralizing antibodies against HIV-1 such as MPER (membrane-proximal external region), CD4 binding site, V1V2 and V3 loop were present on the surface of VSV-GP-Env particles. Binding of quaternary antibodies indicated a trimeric structure of VSV-GP incorporated Env. We detected high HIV-1 antibody titers in mice and showed that vectors expressing membrane-anchored Env elicited higher antibody titers than vectors that secreted Envs. In rabbits, Tier 1A HIV-1 neutralizing antibodies were detectable after prime immunization and titers further increased after boosting with a second immunization. Taken together, VSV-GP-Env is a promising vector vaccine against HIV-1 infection since this vector permits incorporation of native monomeric and/or trimeric HIV-1 Env into a viral membrane.

## 1. Introduction

Human immunodeficiency virus (HIV) infection is still a major health problem with around 36.9 million people living with HIV worldwide [1]. The introduction of the highly active antiretroviral therapy (HAART) for the treatment of HIV-infection has dramatically improved the quality of life and the survival of HIV-infected patients. However, only 46% of HIV-infected individuals received HAART at the end of 2015 and the number of new infections is still high with ~2.1 million per year [1]. As a consequence, HAART, although effectively reducing the incidence of acquired immunodeficiency syndrome (AIDS), has not had a major impact on the global prevalence of HIV infection and ultimately, an effective HIV vaccine will likely be necessary to control the HIV pandemic.

Numerous HIV vaccine strategies have been developed and evaluated in preclinical and clinical studies during the past decades since the discovery of HIV. Since HIV infection inevitably results in a persistent infection, ideal HIV vaccine approaches should aim to induce sterile immunity. It is believed that high titers of broadly neutralizing antibodies (bnAbs) can protect from HIV infection. This was also confirmed in preclinical studies showing that passive infusion of bnAbs results in protection of non-human primates from simian/human immunodeficiency virus (SHIV) [2,3]. However, all HIV vaccine candidates have so far failed to induce bnAbs and in the RV144 HIV vaccine trial, the only clinical trial that showed moderate protection, this protection was correlated with non-neutralizing V1V2-binding antibodies [4,5].

Many successful vaccines for other diseases consist of live-attenuated pathogens, e.g., polio, measles, mumps and rubella. These vaccines confer strong and long-lasting immunity [6]. Since HIV attenuation is not feasible for use in man, viral vector vaccines are an alternative, maintaining the advantages of live-attenuated viral vaccine vectors, such as strong and lasting immune responses and a cost-effective production process while having a greatly enhanced safety profile. One such promising candidate as an HIV vaccine vector is the vesicular stomatitis virus (VSV), an enveloped negative-strand RNA virus. VSV can incorporate HIV Envelope (Env) into its membrane which should have several advantages compared to soluble Env [7]. HIV Env incorporated in the VSV membrane should resemble Env on HIV particles more closely than soluble Env and additionally the membrane is expected to stabilize the Env trimer conformation and to help binding of antibodies against the membrane-proximal external region (MPER) [8]. Soluble gp140 molecules often have deletions of MPER as this hydrophobic domain can lead to protein aggregation [9]. A virus incorporated HIV Env might provide additional benefit as virus particles will be taken up by Env-specific B cells that then present viral epitopes on major histocompatibility complex (MHC) class II molecules. The intrastructural help from virus-specific T helper cells can enhance the production of Env-specific antibodies [10].

The first VSV-based vectors as HIV vaccine have already been explored in the early 2000s and protected non-human primates in SHIV challenge models [11,12,13,14]. However, VSV-based vectors containing the VSV glycoprotein G need massive attenuations in order to achieve a safety profile acceptable for human vaccines as they are neurotropic and can cause neurotoxicity in mouse and primate models [15,16,17]. Such vectors with a shuffled gene order and a C terminally truncated G have been and are currently tested in clinical trials with the aim to either induce T cell responses using Gag or antibody responses using Env [18,19,20]. Another approach to circumvent VSV’s neurotoxicity is to delete the glycoprotein G and functionally replace it with an HIV Env as presented by Christopher Parks on the 18th Annual International Meeting of the Institute of Human Virology [21]. However, production is complicated by the fact that the virus is pseudotyped with VSV-G during production to broaden cell tropism in the first round of in vivo infection. As VSV-G is cytotoxic it is not feasible to generate cell lines stably expressing G and therefore cells need to be transfected or electroporated prior to virus production [22]. In addition, all vectors containing VSV-G have the additional disadvantage that VSV-G induces high titers of vector neutralizing antibodies already a few days after the first immunization which make different serotypes necessary for boosting [23].

We previously described a new variant of VSV, which seems to be an ideal candidate for an HIV vaccine and has several advantages compared to previously described VSV-based HIV vaccine vectors [24]. The glycoprotein G from VSV has been replaced by the full-length glycoprotein GP from the lymphocytic choriomeningitis virus (LCMV) strain HPI. The resulting virus, VSV-GP, is non-toxic in immunocompetent and immunodeficient mice and completely lacks VSV’s inherent neurotoxicity [25,26]. On the other hand, VSV-GP is fully replication competent, can easily be grown to high titers and can infect a broad spectrum of different cell types from many mammalian hosts including mice and humans. Importantly, the virus induces strong and lasting immune responses against a foreign antigen but does not induce vector neutralizing antibodies in mice even after eight repeated applications and, consequently, can be used for homologous boosting [24,27].

Here, we compared VSV-GP vectors encoding different membrane-anchored and secreted variants of the HIV-1 transmitted/founder strain 1086.C Env with regards to the incorporation of Env into the viral particle, Env structure, and immunogenicity of the vectors in mice and rabbits. Membrane-anchored Env fused to a VSV-G C-terminus with a mutated furin cleavage site or a flexible linker showed the highest level of incorporation into the particle in combination with the presentation of several known epitopes for bNAbs, high level of antibody induction in mice and Tier 1A neutralizing antibody induction in rabbits. These VSV-GP-Env vaccine variants are thus highly promising vaccine candidates.

## 2. Materials and Methods

### 2.1. Ethics Statement

Animal experiments were performed in compliance with the national animal experimentation law (“Tierversuchsgesetz”) and animal trial permission was granted by national authorities (Bundesministerium für Wissenschaft und Forschung, #BMWFW-66.011/0139-WF/V/3b/2014, BMWFW-66.011/0148-WF/V/3b/2016 and BMBWF-66.011/0036-V/3b/2018).

### 2.2. Cell Lines

BHK-21 cells (American Type Culture, Manassas, VA, USA) were cultured in Glasgow minimum essential medium (GMEM; Gibco, Carlsbad, CA, USA) supplemented with 10% fetal calf sera (FCS; Gibco, Life Technologies, Carlsbad, CA, USA), 5% tryptose phosphate broth (Gibco, Carlsbad, CA, USA), 100 units/mL penicillin (Gibco, Carlsbad, CA, USA) and 0.1 mg/mL streptomycin (Gibco, Carlsbad, CA, USA). 293T cells (ATCC) and 293T variants expressing LCMV-GP or VSV-G were cultured in Dulbecco’s Modified Eagle’s Medium (DMEM; Lonza Walkerville, MD, USA) supplemented with 10% FCS, 2 mM l-glutamine, 100 units/mL penicillin and 0.1 mg/mL streptomycin. PM-1 cells (obtained through the NIH AIDS Reagent Program, Division of AIDS, NIAID, NIH: PM1 from Dr. Marvin Reitz) were cultured in RPMI Medium 1640 (Gibco, Carlsbad, CA, USA) supplemented with 10% FCS, 2 mM l-glutamine, 100 units/mL penicillin and 0.1 mg/mL streptomycin.

### 2.3. Viruses

**Figure 1:**
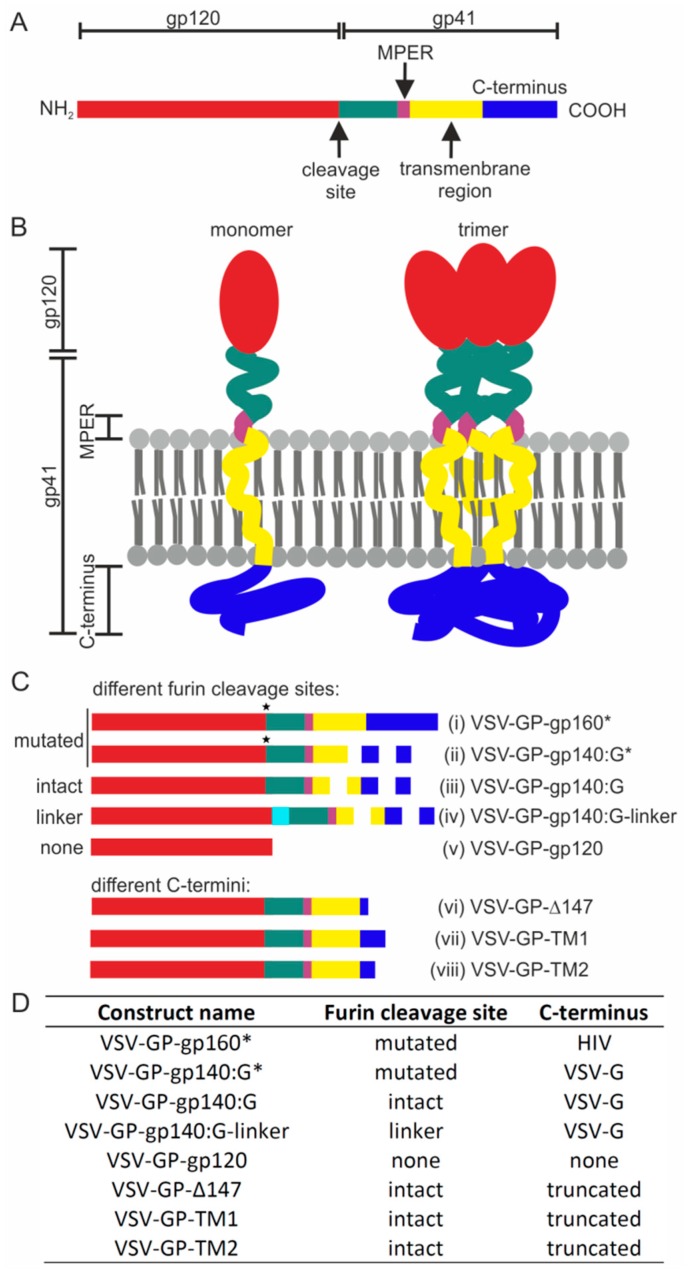
Schematic overview of human immunodeficiency virus (HIV) Env variants. The linear structure of HIV gp160 (**A**) and the three-dimensional structure of the monomers and trimers on the surface of infected cells or viral particles (**B**) are shown. (**C**) Schematic overview of HIV Env variants that were cloned into position 5 of VSV-GP vectors. Color coding: gp120 (red), extracellular part of gp41 (green), MPER (membrane-proximal external region; purple), transmembrane domain (TM; solid line for HIV-TM or dotted line for vesicular stomatitis virus glycoprotein (VSV-G)-TM, yellow) and C-terminus (solid line for HIV C-terminus or dotted line for VSV-G C-terminus, dark blue). Constructs contained either a functional furin cleavage site, a mutated furin cleavage site (indicated by an asterisk) or a flexible linker replacing the furin cleavage site (light blue). (i) gp160* = full-length HIV Env with a mutated furin cleavage site, (ii) gp140:G* = gp140 fused to the transmembrane domain and cytoplasmic tail of VSV-G with a mutated furin cleavage site, (iii) gp140:G = gp140 fused to the transmembrane domain and cytoplasmic tail of VSV-G with an intact furin cleavage site, (iv) gp140:G-linker = gp140 fused to the transmembrane domain and cytoplasmic tail of VSV-G with a flexible linker replacing the furin cleavage site, (v) gp120, (vi) Δ147 = truncated HIV Env with only 3 intracellular amino acids at the C-terminus, and an intact furin cleavage site, (vii) TM1 = HIV Env with an SIV segment insertion at the C-terminus, a Y712I mutation, a STOP codon after F717, and a native furin cleavage site, and (viii) TM2 = HIV Env with the SIV segment insertion, a deletion of GY711-712, R722G and S727P mutations (SIV_mac239_ numbering), a STOP codon after F717, and an intact furin cleavage site. (**D**) Furin cleavage site configuration and C-terminus for all constructs are depicted.

VSV-GP and VSV-GP-OVA were described previously [24,25]. HIV Env constructs were added into a VSV-GP variant, containing an additional intergenic region on position 5 between GP and L, [26] via NheI and XhoI restriction sites. A schematic overview of VSV-GP-Env variants is shown in Figure 1. Virus rescue was done on 293T cells via reverse genetics with a helper virus-free protocol as described before [28]. Newly rescued viruses were passaged on BHK-21 cells and plaque purified twice on BHK-21. Virus stocks were produced on BHK-21 cells, concentrated via overnight low-speed centrifugation using a 20% sucrose cushion and titrated via 50% tissue culture infective dose (TCID_50_) assay. Replication-defective VSV*ΔG-gp140:G* virus was generated by deleting the LCMV-GP in VSV-GP-gp140:G*. Newly rescued viruses were passaged and plaque purified on BHK-21 cells expressing LCMV-GP.

### 2.4. TCID_50_ Assay

Viral titers were determined via a TCID_50_ assay as described by Spearman-Kaerber [29]. Briefly, 1 × 10^3^ BHK-21 cells were seeded per well of a 96-well plate and incubated overnight at 37 °C. One hundred microliter of a 10-fold serial dilution of the virus was added to the cells in 8 replicates. After 6 days of incubation at 37 °C, wells with a visible cytopathic effect were counted.

### 2.5. Replication Kinetics

BHK-21 cells were seeded in 24-well plates with 1 × 10^5^ cells per well. After overnight incubation, cells were infected with an MOI of 0.1 in duplicates. Cells were incubated for 1 h at 37 °C and afterwards washed twice with PBS. Fresh medium was added and supernatants were collected after 4, 8, 24 and 48 h and viral titers were determined by TCID_50_ assay.

### 2.6. Preparation of Lysates and Western Blot Analysis

BHK-21 cells were infected with an MOI of 0.1, incubated at 37 °C for 24 h and subsequently lysates were prepared as previously described [24]. Uninfected BHK-21 cells were used as a negative control. Purified viruses were diluted in PBS to a concentration of 3.16 × 10^8^–3 × 10^9^ (TCID_50_ per sample) and both, cell lysates or viruses, were analyzed under standard non-reducing conditions on 10% polyacrylamide gels as described before [24]. For staining of membranes HIV gp41-specific (4E10, Polymune, Klosterneuburg, Austria), HIV gp120-specific (16H3, kindly provided through the NIH AIDS Reagent Program, Division of AIDS, NIAID, NIH by Dr. Barton Haynes and Dr. Hua-Xin Liao) or VSV-N-specific (Kerafast, Boston, MA, USA) antibodies were used in combination with HRPO-conjugated secondary antibodies.

### 2.7. Flow Cytometry Analysis

For staining of cells, BHK-21 cells were infected with an MOI of 0.1 of VSV-GP-Env variants or VSV-GP as a negative control. After overnight incubation, cells were trypsinized and washed once with FACS buffer (PBS supplemented with 1% FCS and 0.05% sodium azide). Prior to staining of LCMV-GP, cells were fixed with 1.5% formaldehyde. Subsequently, cells were stained with the LCMV-GP-specific antibody WEN4 (kindly provided by Annette Oxenius, ETH Zurich, Switzerland) and a fluorescently-labeled secondary anti-mouse antibody. For staining of HIV Env, HIV bnAbs (kindly provided by IAVI (PG9, PG16, PGT121), Dr. Xueling Wu, Dr. Zhi-Yong Yang, Dr. Yuxing Li, Dr. Gary Nabel, Dr. John Mascola (VRC01), Dr. Dennis Burton and Dr. Carlos Barbas (b12), Dr. Michel C. Nussenzweig (3BNC117), Dr. James E. Robinson (39F), Duke Human Vaccine Institute, Duke University Medical Center (CH106) through the NIH AIDS Reagent Program, Division of AIDS, NIAID, NIH) and a fluorescently-labeled secondary anti-human antibody were used.

For the staining of viruses, the purified virus was coupled to Adju-Phos^®^ (a kind gift by Brenntag Biosector A/S, Ballerup, Denmark, 10 µL of a 1:100 dilution per sample) for 30 min at 37 °C. Samples were washed once with PBS and subsequently, the remaining free binding sites were blocked with 3% bovine serum albumin for 30 min at 37 °C. Antibody staining was performed analogously to cell samples. Samples were fixed with 1.5% formaldehyde and measured using a FACS Canto II and DIVA software (version 6.1.3, Becton Dickinson, Vienna, Austria). Samples were analyzed using FlowJo software (version 10, FlowJo, LLC, Ashland, OR, USA).

### 2.8. Electron Microscopic Pictures

Viruses were produced on BHK-21 cells and supernatants were concentrated via overnight low speed centrifugation with a 20% sucrose cushion. Virus pellets were resuspended in PBS and paraformaldehyde (8%) was added to a final concentration of 4% to inactivate viruses. Samples were stored at −80 °C till analysis. Viruses were adsorbed to the clean side of a carbon film on mica, stained with 2% PTA pH 7.0 or 2% AmMo pH 7.4, attached to a 400-mesh copper grid and transferred into an FEI F20 electron microscope operating at 200 kV. The images were taken on a 4 k by 4 k Gatan OneView camera at nominal magnifications of 80,000- or 100,000-fold.

### 2.9. Mouse Immunization Experiments

Female C57BL/6J wild type mice were purchased from Janvier (Le Genest-Saint-Isle, France) and maintained in the animal facilities of the Innsbruck Medical University. Mice (8 per group) were immunized intramuscularly with 1 × 10^7^ TCID_50_ of VSV-GP-Env constructs diluted in PBS at weeks 0, 4, and 8. Control animals received immunizations with VSV-GP. EDTA-blood was collected prior to the first immunization and 4 weeks after each immunization. Plasma was prepared and stored at −80 °C.

### 2.10. Rabbit Immunization Experiments

Female New Zealand White rabbits were purchased from Charles River (Sulzfeld, Germany) and maintained in the animal facilities of the Innsbruck Medical University. Groups of 4 animals were immunized intramuscularly at weeks 0, 3, and 6 with 2 × 10^8^ TCID_50_. Prior to the first immunization and 3 weeks after each immunization blood was collected. Blood was allowed to coagulate for 1 h at room temperature and serum was prepared afterwards. Serum was stored at −80 °C.

### 2.11. Anti-1086.C-gp140 Enzyme-Linked Immunosorbent Assay (ELISA)

96-well Maxisorp plates (Nunc, Fisher Scientific, Vienna, Austria) were coated with 50 ng/well of a 1086.C gp140 protein [30] in 0.2 M Na_2_CO_3_- NaHCO_3_, pH 9.5, overnight at 4 °C. Blocking was performed with superblock (PBS with 4% Whey protein, 15% goat serum, 0.5% Tween20) for 1 h at 4 °C. All washing steps were performed with PBS containing 0.1% Tween20 (PBST). Mouse and rabbit plasma samples were heat inactivated for 1 h at 56 °C and pre-diluted in superblock 1:100. Samples were further serially diluted 1:3-fold and transferred in duplicates to the pre-coated 96-well plate and incubated for 1–1.5 h at room temperature. Detection was performed using a horseradish peroxidase conjugated mouse or rabbit IgG-specific antibody from goat (incubation of a 1:10,000 dilution for 1 h at 37 °C) and Sure Blue TMB detection reagent and TMB stop solution (KPL, Gaithersburg, MD, USA). Plates were analyzed at 450 nm (signal) and 650 nm (background) on a model 680 microplate reader (Bio-Rad, Hercules, CA, USA) using Microplate Manager 5.2.1 software (Bio-Rad, Hercules, CA, USA). Endpoint titers were determined as the reciprocal maximum dilution at which the mean optical density (OD) at 450 nm minus the background (OD450–OD650) of duplicates was greater than the mean OD450–OD650 plus 3 standard deviations of naive sera, the double mean of naive sera, and 0.1 for reliable values.

### 2.12. HIV Neutralization Assays

Neutralizing antibodies were determined in a TZM-bl assay as described before [31].

### 2.13. Statistical Analysis

Comparisons of the three groups were performed by the Kruskal-Wallis test. If this test resulted in a 2-sided *p*-value < 0.05, then two 2-sided pairwise Wilcoxon Rank Sum tests with Holm’s adjustment for multiple comparisons were performed (as a non-parametric version of Dunn’s test). This analysis was done separately for the vaccine prime, 1st boost, and 2nd boost results.

## 3. Results

### 3.1. Membrane-Anchored Env Induces Higher Antibody Titers Compared to Secreted Env

To induce HIV neutralizing antibodies, we aimed to generate VSV-GP particles that displayed HIV Env on the surface of the viral particles as trimers in a native conformation. For this purpose, we generated a set of VSV-GP-Env variants (Figure 1) and analyzed the conformation of the Env on infected cells and viral particles.

**Figure 2:**
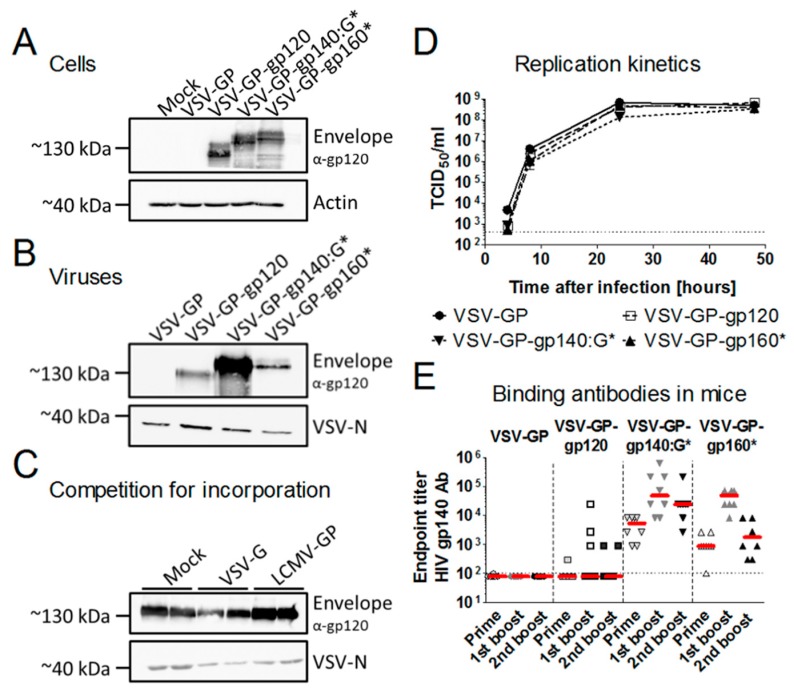
Incorporation of HIV Env into VSV-GP particles improved antibody responses. (**A**) BHK-21 cells were infected with VSV-GP, VSV-GP-gp120, VSV-GP-gp160* or VSV-GP-gp140:G* at an multiplicity of infection (MOI) of 0.1. 24 h post-infection, cell lysates were prepared. (**B**) VSV-GP, VSV-GP-gp120, VSV-GP-gp160* or VSV-GP-gp140:G* were produced and concentrated via a sucrose cushion. Cell lysates (**A**) or viral particles (**B**) were analyzed via western blotting with a gp120-specific antibody (16H3). As a loading control, actin- or VSV-N-specific antibodies were used. (**C**) 293T cells (Mock), 293T cells expressing VSV-G or 293T cells expressing lymphocytic choriomeningitis virus glycoprotein (LCMV-GP) were infected in duplicates with the replication-defective VSV*ΔG-gp140:G* at an MOI of 0.2. The supernatant was collected 24 h later and viruses were concentrated via low-speed overnight centrifugation through a sucrose cushion. Western blotting was performed with purified viruses using an anti-HIV-1 gp120 monoclonal antibody (16H3) to detect HIV Env. A monoclonal antibody against VSV-N was used as loading control. (**D**) BHK-21 cells were infected with VSV-GP, VSV-GP-gp120, VSV-GP-gp160* or VSV-GP-gp140:G* at an MOI of 0.1 in duplicates. Four, 8, 24, and 48 h after infection, the supernatant was collected and analyzed for viral titer via TCID_50_ (50% tissue culture infective dose) assay. Mean ± SEM are shown. The dotted line shows the detection limit of the assay. (**E**) C57BL/6 mice (*n* = 8) were immunized intramuscularly with 10^7^ TCID_50_ of VSV-GP, VSV-GP-gp120, VSV-GP-gp160* or VSV-GP-gp140:G* at weeks 0, 4, and 8. Four weeks after each immunization, plasma was collected and analyzed for the titer of gp140-binding antibodies via ELISA. The median for each group (red line) and the titer for each individual animal is shown. The dotted line shows the detection limit of the assay.

Previously, it had been shown by others that full-length HIV Env is only poorly integrated into the wild-type VSV envelope, but that truncated or Env/VSV-G-C-term chimeric proteins can be efficiently incorporated [7,11,12]. Therefore, we constructed an Env/VSV-G chimeric variant where the extracellular domain of the HIV Env with a mutated furin cleavage site was fused to the transmembrane domain and the cytoplasmic tail from VSV-G. We analyzed expression in infected cells and incorporation into VSV-GP particles for this chimeric protein compared to full-length HIV Env with a mutated furin cleavage site and a secreted gp120. As expected, VSV-GP behaved similarly compared to wild-type VSV. Although all three proteins were expressed at similar levels in VSV-GP-Env infected cells, only Env/VSV-G chimeric protein was efficiently incorporated into viral particles (Figure 2A,B). While in the cell lysates different Env variants, representing most likely intracellular glycosylation variants during protein production, were detected, only one band was found in virus particles.

Next, we analyzed whether expression of LCMV-GP influenced HIV Env incorporation. Replication-defective VSV*ΔG-gp140:G* particles were either produced on cells not expressing an additional glycoprotein, on cells expressing VSV-G or on cells expressing LCMV-GP. The amount of HIV Env in the viral particles was not different among viruses produced on the three different cell lines (Figure 2C). Additionally, we asked whether incorporation of HIV Env into VSV-GP particles might attenuate VSV-GP. To answer this question, we performed replication kinetics comparing replication of VSV-GP (without an additional antigen), VSV-GP-gp120 (with a secreted Env), VSV-GP-gp160* (with a weak incorporation of Env into the particles, * indicates a mutated furin cleavage site) and VSV-GP-gp140:G* (with a strong incorporation of Env into the particles). All viruses had similar replication kinetics (Figure 2D). Both experiments indicated that neither LCMV-GP nor VSV-G influenced the incorporation of HIV Env into the viral envelope.

Next, we sought to determine whether incorporation of HIV Env into the vaccine vector can improve antibody responses as we hypothesized. To do so, we immunized mice either with a VSV-GP vector with high incorporation of Env (VSV-GP-gp140:G*), with poor incorporation (VSV-GP-gp160*) or with a secreted gp120 (VSV-GP-gp120). All immunizations were done without adjuvants as we used infectious, replication-competent VSV-GP-Env variants. Plasma was collected four weeks after prime or boost immunizations and analyzed for titers of gp140-specific antibodies. Antibody titers were highest for the construct with the virus incorporated Env, VSV-GP-gp140:G* (Figure 2E), while both membrane-anchored constructs elicited higher Env-specific antibody titers than the secreted gp120. A Kruskal-Wallis test followed by two 2-sided pairwise Wilcoxon Rank Sum tests were done after each immunization. Indeed, as expected antibody titers were significantly higher for VSV-GP-gp160* compared to VSV-GP-gp120 at all time points and VSV-GP-gp140:G* titers were significantly higher than VSV-GP-gp160* titers after prime and second boost immunization (Supplementary Table S1).

### 3.2. HIV Env is Expressed on the Surface of Infected Cells

Therefore, in the next set of constructs, we aimed to further enhance incorporation and improve the structure of Env trimers presented on the virus particle. As cleavage of the gp160 precursor protein can influence folding of HIV Env and lead to the shedding of gp120 protein, we cloned three Env/VSV-G constructs with different configurations of the furin cleavage site. The variants VSV-GP-gp140:G* and VSV-GP-gp140:G contained a mutated (*) and intact furin cleavage site, respectively. In the virus VSV-GP-gp140:G-linker the furin cleavage site was replaced by a 10 amino acid flexible linker that has been reported to support the native trimer conformation of Env [32]. Additionally, we generated the variant VSV-GP-Δ147 with a truncated cytoplasmic tail, leaving only three intracellular amino acids, and the two variants VSV-GP-TM1 and VSV-GP-TM2. The latter two variants have been described to be incorporated into lentiviral vector particles better than full-length Env [33,34]. The TM1 construct contained an SIV segment insertion at the C-terminus, a Y712I mutation, and a STOP codon after F717. The TM2 construct contained an SIV segment, a deletion of GY711-712, R722G and S727P mutations (SIV_mac239_ numbering), and a STOP codon after F717. The later three constructs have an intact furin cleavage site. All HIV *env* variants were cloned into position 5 as an additional transgene into the VSV-GP genome. Infectious viruses were generated via reverse genetics and the sequence of the HIV gene inserts was verified.

**Figure 3:**
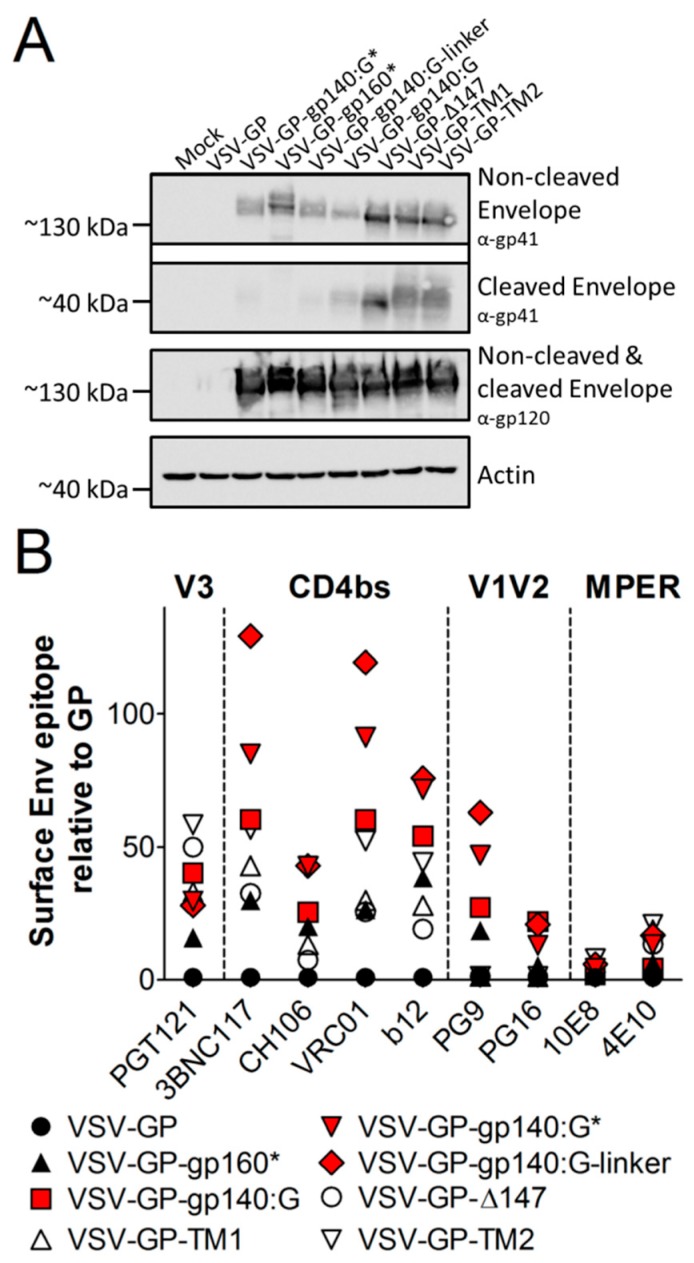
All HIV Env variants are expressed at comparable levels in infected BHK-21 cells. BHK-21 cells were infected with VSV-GP, VSV-GP-gp140:G*, VSV-GP-gp160*, VSV-GP-gp140:G-linker, VSV-GP-gp140:G, VSV-GP-Δ147, VSV-GP-TM1 and VSV-GP-TM2 with a MOI of 0.1. Twenty-four hours after infection, cell lysates were prepared for western blotting (**A**) or cells were trypsinized and analyzed by flow cytometry (**B**). As a negative control, cell lysates from non-infected BHK-21 cells (Mock) were used. (**A**) The non-cleaved HIV Env precursor protein (gp160) and the cleaved gp41 protein were detected with an anti-HIV-1 gp41 monoclonal antibody (4E10, upper two blots). The non-cleaved Env precursor and the cleaved gp120 subunit were detected on a separate membrane using an anti-HIV-1 gp120 monoclonal antibody (16H3, third blot). As a loading control, an anti-actin antibody was used (lower blot). (**B**) Cells were stained with HIV bnAbs against the V1V2 loop of gp120 (PG9, PG16), the V3 loop of gp120 (PGT121), the CD4 binding site (b12, VRC01, 3BNC117, CH106) or the MPER of gp41 (4E10) and fluorescently-labeled anti-human secondary antibodies. As a loading control, the LCMV-GP-specific WEN4 was used. The ratio of the geometric mean fluorescence of every single HIV-specific antibody relative to the geometric mean fluorescence of the LCMV-specific WEN4 was calculated. The graph shows the mean ratio of duplicate samples for each antibody and virus.

First, we analyzed the expression of the different Env variants in VSV-GP-Env infected BHK-21 cells (Figure 3A). For detection, we used two different antibodies, one binding to gp41 (4E10) for detection of non-cleaved gp160 and cleaved gp41 and the second one against gp120 (16H3) for detection of non-cleaved gp160 and cleaved gp120. All Env variants were expressed in VSV-GP-Env infected cells at comparable levels. For the four variants with an intact furin cleavage site (VSV-GP-gp140:G, VSV-GP-Δ147, VSV-GP-TM1 and VSV-GP-TM2) cleaved gp41 was detected.

In the next step, we performed flow cytometry analysis to see if HIV Env was also transported to the cell surface and to analyze folding of the Env variants. As the V1V2 loop, the V3 loop, the CD4 binding site (CD4bs) and MPER are important epitopes for binding of HIV bnAbs and display of these epitopes might also be favorable in a vaccine for induction of bnAbs, we analyzed if Env on the surface displayed these epitopes. Therefore, we choose a set of bnAbs that bind to the V1V2 loop of gp120 (PG9, PG16), the V3 loop of gp120 (PGT121), the CD4 binding site (b12, VRC01, 3BNC117, CH106) or the MPER of gp41 (4E10). All Env variants were expressed on the surface of infected cells (Figure 3B). It is interesting that binding of HIV bnAbs differed among the variants. The three Env/VSV-G chimeric variants (VSV-GP-gp140:G*, VSV-GP-gp140:G and VSV-GP-gp140:G-linker) were nicely detected by most antibodies (red symbols in Figure 3B). It is of note that VSV-GP-gp140:G* and VSV-GP-gp140:G-linker were recognized best by antibodies binding to quaternary epitopes and therefore preferentially to intact trimers (PG9).

### 3.3. HIV Env on the Surface of VSV-GP Particles Displays Crucial Epitopes for Binding of Neutralizing Antibodies

**Figure 4:**
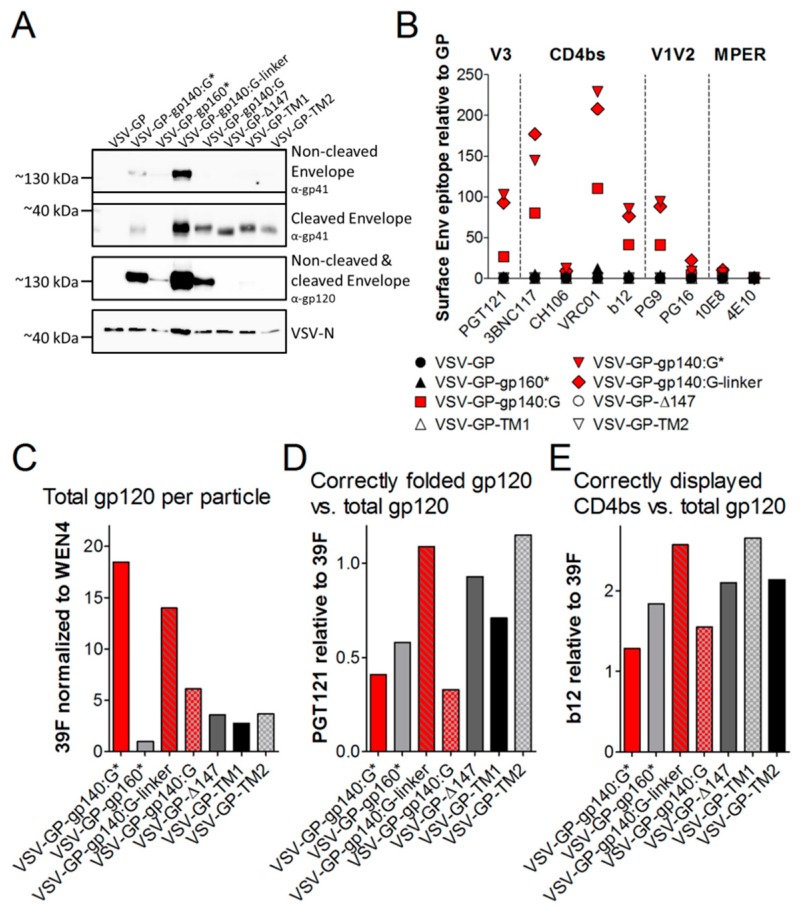
HIV Env is incorporated into VSV-GP particles and displays epitopes crucial for binding of bnAbs. The different VSV-GP particles (VSV-GP, VSV-GP-gp140:G*, VSV-GP-gp160*, VSV-GP-gp140:G-linker, VSV-GP-gp140:G, VSV-GP-Δ147, VSV-GP-TM1 and VSV-GP-TM2) were produced on BHK-21 cells and concentrated via low-speed centrifugation using a sucrose cushion. (**A**) Purified particles were diluted in PBS to a concentration of 10^9^ TCID_50_/mL and used for western blot analysis. The non-cleaved HIV Env precursor protein gp160 and the cleaved gp41 protein were detected by using an anti-HIV-1 gp41 monoclonal antibody (4E10, upper two blots). The non-cleaved Env precursor gp160 and the cleaved gp120 subunit were detected on a second membrane using an anti-HIV-1 gp120 monoclonal antibody (16H3, third blot). As a loading control, a monoclonal antibody against VSV-N was used (lower blot). (**B**–**E**) Purified particles were diluted in PBS to a concentration of 10^9^ TCID_50_/mL and used for flow virometry analysis. Forty microliter of virus dilution (corresponding to 4 × 10^7^ TCID_50_) was used per sample. The virus was complexed with Adju-Phos^®^. (B) Samples were stained with bnAbs against the V1V2 loop of gp120 (PG9, PG16), the V3 loop of gp120 (PGT121), the CD4 binding site (b12, VRC01, 3BNC117, CH106) or the MPER of gp41 (4E10) and fluorescent labeled anti-human secondary antibodies. As a loading control, the LCMV-GP-specific antibody WEN4 with a fluorescent labeled anti-mouse secondary antibody was used. The ratio of the geometric mean fluorescence for every single HIV-specific antibody relative to the geometric mean fluorescence of the LCMV-specific WEN4 was calculated. The graph shows the mean ratio of duplicate samples for each antibody and virus. (**C**) Samples were stained with antibodies recognizing a linear V3 epitope (39F) or the LCMV-GP-specific antibody WEN4. Geometric mean fluorescence of 39F stainings (gp120 on virus particles) was normalized to WEN4 geometric mean fluorescence (loading of viruses to beads). The graph shows the mean of duplicate samples. (**D**–**E**) Viruses were stained with the conformation-dependent V3-specific antibody PGT121, CD4bs-specific b12, the linear V3-specific 39F or the LCMV-GP-specific WEN4. Geometric mean fluorescence of Env-specific antibodies was normalized to the loading of the virus using the geometric mean fluorescence of WEN4. To analyze the ratio of correctly folded versus total Env on the virus the ratio of PGT121/39F (**D**) or b12/39F (**E**) was calculated.

As we hypothesized that incorporation of HIV Env into VSV-GP particles will increase Env-specific antibody responses via intrastructural help [10] compared to a secreted Env or an Env expressed only on the surface of infected cells, we next analyzed incorporation of HIV Env into VSV-GP particles for the whole set of constructs as shown in Figure 1C. Viruses were pelleted through a sucrose cushion to remove shed Env. Purified viruses were analyzed with gp41- or gp120-specific antibodies to detect non-cleaved Env/cleaved gp41 or non-cleaved Env/cleaved gp120 respectively. Although all Env constructs were similarly expressed in infected cells, we observed strong differences in incorporation into VSV-GP particles (Figure 4A). As seen before for the construct with the mutated furin cleavage site, we saw much better incorporation also for the two other Env/VSV-G chimeric proteins with an intact furin cleavage site or the flexible linker replacing the cleavage site compared to full-length Env into VSV-GP. Additional truncation of the cytoplasmic tail (Δ147) or the TM-configurations did not improve incorporation compared to gp140:G. The two TM constructs and the Δ147 construct showed only a clear band for cleaved gp41, indicating that the Env in these three constructs was completely cleaved and that gp120 was shed. In contrast, the gp140:G construct, which also contained an intact furin cleavage site, was not shed although it was also completely cleaved. Non-cleaved Env was only detected for constructs with a mutated furin cleavage site or the linker replacing it. Surprisingly, VSV-GP-gp140:G* showed a weak and VSV-GP-gp140:G-linker a strong signal at the size of cleaved gp41 which might be explained by degradation products or the use of cryptic cleavage sites.

As viruses with cleaved Env on the surface might infect and subsequently kill CD4^+^ cells, we analyzed infectivity of our viruses on the CD4^+^ T cell line PM-1. The parental VSV-GP did not infect PM-1 cells via LCMV-GP. Although cells for the VSV-GP-Env virus variants were infected at an MOI of 10 as determined on BHK-21 cells no infection of the PM-1 cells was detected via FACS analysis.

When analyzing the conformation of the HIV Env on the surface of VSV-GP particles, we got similar results as determined for infected cells (Figure 4B). The three HIV Env/VSV-G chimeric variants were incorporated well into the VSV-GP particles and especially the linker construct strongly displayed all epitopes necessary for binding of the tested HIV bnAbs (red symbols in Figure 4B). As western blot analysis had shown that full-length HIV Env was not well incorporated into the VSV-GP particles, it was not surprising that in the flow virometry staining the signal was low for all antibodies, except the LCMV-GP-specific WEN4 that served as loading control. In concordance with western blot analysis, the low signals in the flow virometry analysis also suggested that cleaved gp120 was shed for the Δ147, the TM1 and the TM2 constructs. To analyze the presentation of gp120 on the viral particles independent of its conformation we also stained VSV-GP-Env particles with the antibody 39F recognizing a linear V3 epitope. As already seen in the western blot this flow virometry analysis confirmed that Env/VSV-G chimeric proteins were incorporated much better into VSV-GP particles compared to full-length gp160 (Figure 4C). Within the three chimeric proteins, the constructs with a mutated furin cleavage site or the linker showed more gp120 on the VSV-GP particle than the one with the intact furin cleavage site, indicating partial shedding of gp120 for the VSV-GP-gp140:G construct. For the Δ147, the TM1 and the TM2 constructs again an even stronger shedding of gp120 was confirmed. To analyze conformation of Env independent of the amount of Env incorporated into the particles we normalized stainings for the conformation-dependent PGT121 (V3 loop) or b12 (CD4bs) to total gp120 on the particle (39F staining). Again the VSV-GP-gp140:G-linker construct showed a very natural folding (Figure 4D,E).

We also analyzed VSV-GP-gp140:G*, VSV-GP-gp140:G and VSV-GP-gp140:G-linker particles via electron microscopy (Figure S1). As a control, VSV-GP-OVA particles were analyzed, which contain the ovalbumin fused to GFP, a non-membrane protein with a size comparable to the gp140:G, at position 5 as an additional transgene. All viruses had the VSV typical bullet-like shape with comparable sizes. For VSV-GP-OVA, individual glycoproteins were seen on the surface of the particle (upper left pictures in Figure S1). In contrast to this, all three virus variants containing HIV Env showed a denser glycosylation pattern on the surface, again indicating strong incorporation of the HIV Env/VSV-G chimeric proteins into the virus membrane.

### 3.4. VSV-GP-Env Induces gp140-Specific Antibodies in Mice and Rabbits

**Figure 5:**
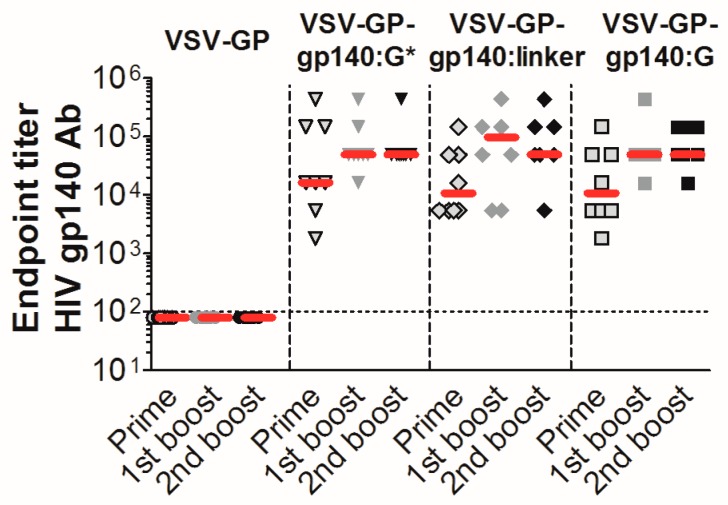
VSV-GP-Env particles induce high titers of gp140 binding antibodies in mice. Groups of 8 C57BL/6 mice were immunized intramuscularly with 10^7^ TCID_50_ of VSV-GP, VSV-GP-gp140:G*, VSV-GP-gp140:G or VSV-GP-gp140:G-linker at week 0, 4, and 8. Four weeks after each immunization, plasma was collected and analyzed for the titer of gp140-binding antibodies using an ELISA (enzyme-linked immunosorbent assay). The median for each group (red line) and the titer for each individual animal are shown. The dotted line shows the detection limit of the assay.

The three Env/VSV-G chimeric proteins were well incorporated into VSV-GP particles, displayed epitopes that are crucial for binding of HIV bnAbs did not or only partially shed gp120, and seemed to form trimers on the surface of infected cells/viral particles. Therefore, we selected these variants for in vivo immunization experiments in mice. As a negative control, mice were immunized with “empty” VSV-GP without an additional transgene. In none of the VSV-GP control vector immunized mice, Env-specific antibodies were induced. In contrast, all VSV-GP-Env vectors induced gp140-specific antibodies already after the first immunization (Figure 5). The induction of humoral immune responses among the different viruses was similar; no significant differences were detected by the Kruskal-Wallis test (Supplementary Table S2).

As HIV neutralizing antibodies are difficult to determine in mouse plasma, we tested the two most promising viruses VSV-GP-gp140:G* and VSV-GP-gp140:G-linker for induction of HIV neutralizing antibodies in rabbits. New Zealand White rabbits were immunized three times with the two viruses intramuscularly with a three-week interval between each immunization. After virus injection, animals showed a mild weight loss or delayed weight gain for a few days and an increased temperature for the first hours after injection. We did not observe any further adverse effects.

**Figure 6:**
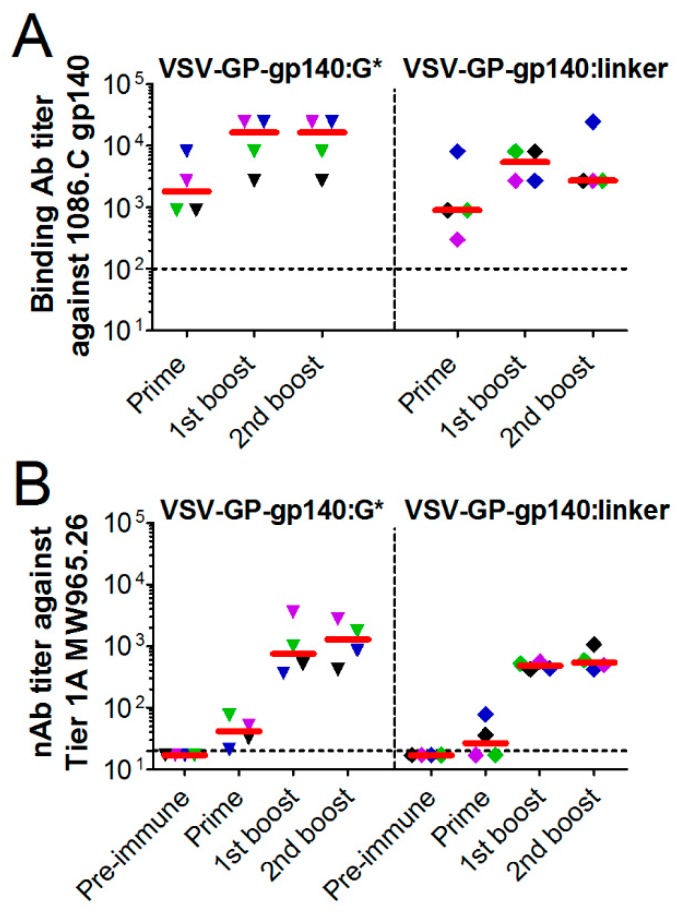
VSV-GP-Env induces gp140 binding antibodies upon immunization of rabbits. New Zealand White rabbits (*n* = 4) were immunized intramuscularly with 2 × 10^8^ TCID_50_ of VSV-GP-gp140:G* or VSV-GP-gp140:G-linker at week 0, 3, and 6. (**A**) Prior to the first immunization and three weeks after each immunization plasma was collected and analyzed for the titer of gp140-binding antibodies using an ELISA. (**B**) Serum samples prior to the first immunization and three weeks after each immunization were analyzed for the titer of neutralizing antibodies against the Clade C Tier 1A HIV-1 MW965.26 virus using a TZM-bl neutralization assay. The median for each group (red line) and the titer for each individual animal are shown. The same colored symbol is used for each animal at different time points. The dotted line shows the detection limit of the assay.

Plasma samples collected three weeks after each immunization were analyzed for gp140-binding antibody titers via ELISA (Figure 6A). Pre-immunization plasma samples were used as negative control. In all animals, gp140-binding antibodies were induced. The antibody titer increased after the first booster immunizations (median 1800 vs. 16,200 for VSV-GP-gp140:G* and 900 vs. 5400 for VSV-GP-gp140:G-linker) but was not clearly different in pattern between the two viruses. We further analyzed serum samples, collected prior to the first and three weeks after each immunization, for HIV neutralizing antibodies in vitro using a pseudovirus assay on TZM-bl cells. We included a Tier 1A Clade C virus, three Tier 2 Clade C viruses (including the 1086.C immunogen strain) and one Tier 2 Clade B virus. As a control, virus pseudotyped with the MLV Env as an indicator of the non-HIV-specific signal was used. We detected HIV neutralizing antibodies in none of the pre-immunization samples. However, we detected neutralizing antibody activity against the Tier 1A Clade C virus already after the prime immunization that was further markedly increased after the first boost (18-fold increase in median neutralizing antibody titers between prime and first boost for VSV-GP-gp140:G* and 20-fold increase for VSV-GP-gp140:G-linker, Figure 6B and Supplementary Table S3). However, the magnitude of responses did not further increase with the second boost. We detected no significant neutralization of Tier 2 viruses in this study (Supplementary Table S3).

## 4. Discussion

Using the model antigen ovalbumin, we previously have shown that VSV-GP is an excellent vaccine vector that can be used for homologous boosting [24]. In the present study, we evaluated VSV-GP as an HIV vaccine vector expressing Env that potentially can induce a protective humoral immune response against HIV.

Entry of HIV into susceptible primate target cells is mediated by the trimeric viral membrane-incorporated Env [35]. One strong advantage of VSV-based vectors is that HIV Env can be incorporated into viral particles in its natural trimeric conformation as observed on HIV-1. We hypothesized that incorporation of HIV Env into the envelope of VSV-GP is an advantage for neutralizing antibody induction. We, therefore, initially tested several Env variants for incorporation into the VSV-GP membrane. As shown for wild-type VSV, we found that full-length HIV Env was not efficiently incorporated into the viral particle. Johnson and colleagues showed that a sequence in the C-terminus of Env prevents efficient incorporation into VSV particles as this sequence redirects Env away from the budding site of VSV [36]. Although the Δ147, TM1 and TM2 variants were incorporated better into lentiviral particles as full-length Env [33], their incorporation into VSV-GP particles was only moderate, which might be due to an incorrect localization in the plasma membrane. In contrast, the Env/VSV-G chimeric proteins with the cytoplasmic tail of VSV-G were incorporated efficiently into the membrane of VSV-GP as also seen by others for wild-type VSV [7,36]. Incorporation of HIV Env and LCMV-GP did not interfere with each other and HIV Env within the viral particle did not limit the replication fitness of the virus.

We then confirmed our predictions that membrane-anchored Env induced higher Env-specific antibody titers than secreted Env and that virus-incorporated Env induced antibodies more efficiently than inefficiently incorporated full-length gp160 or secreted Env. Alexander and colleagues showed for a replicating Ad4 vector that membrane-anchored Env induces better immune responses than secreted Env [37], similarly as we saw for VSV-GP. However, the second part of our findings cannot be confirmed in an adenoviral vector as this virus has no membrane to incorporate HIV Env. Indeed, the incorporation of Env into the viral membrane is a special feature for VSV-GP and only a few other vaccine vectors allow efficient incorporation of HIV Env into their membrane. Apart from VSV also other negative-strand RNA viruses such as Newcastle disease virus allow incorporation of HIV Env into their membrane [38]. In contrast to this, poxviruses, another enveloped virus family and one of the gold standards for HIV vaccine development, have a very complex replication cycle with several stages involved. For vaccinia virus, it has been shown that HIV Env is mainly found within the protein-DNA-fraction but not within the envelope fraction [39]. Thus the possibility to present Env on the surface of the viral particle and consequently induce increased antibody titers recommends VSV-GP as an ideal HIV vaccine vector.

There are several possible explanations for the improved immunogenicity of membrane-anchored or particle-incorporated Env. Bachmann and colleagues showed for VSV-G that densely packed and repetitively organized glycoproteins produce better antibody responses compared to poorly organized glycoprotein in solution [40]. In addition, membrane-anchored Env might have a longer half-life compared to secreted Env. Finally, intrastructural help is expected to augment antibody induction against particle-associated Env [10,41]. Previous studies indicated that viral particles, which contain Env on the surface, are taken up by Env-specific B cells via the B cell receptor. These B cells then present peptides derived from all viral proteins via MHC-II and thereby induce a potent Th2 helper response that in turn supports expansion and differentiation of Env-specific B cells.

In the next step, we analyzed the folding of the virus-incorporated Env. Most bnAbs target tertiary or quaternary structures and Envs used for vaccination should, therefore, fold into a native conformation. Indeed, we could show that the Env/VSV-G chimeric variants with a mutated furin cleavage site and with a flexible linker replacing the furin cleavage site highly resembled the native Env conformation. All broadly neutralizing antibodies tested efficiently bound to Env/VSV-G chimeric Envs on VSV-GP particles. It is interesting that the VSV-GP-gp140:G-linker construct was recognized best by the V1V2 binding antibodies. In the RV144 trial, the only HIV vaccine trial that showed some efficacy so far, V1V2 binding, non-neutralizing antibodies were positively correlated with protection [4].

Sharmar and colleagues showed that the conformation of soluble Env proteins with a flexible linker replacing the furin cleavage site highly resembles the native Env conformation [32]. Their constructs are similar to the linker construct we used here. We did not explore the so-called SOSIP conformation of Envs [42]. SOSIPs are cleaved gp140 molecules held together by an additional disulfide bridge between both subunits. Previous studies showed that soluble, stabilized SOSIP trimers induce better antibody responses than uncleaved gp140 [43]. However, SOSIPs need to be furin cleaved for correct folding. For our VSV-GP vector not all in vivo target cells might express sufficient amounts of furin which might ultimately result in improper Env folding. Both strategies, SOSIP and native-flexible linker, induced as protein vaccine similar neutralizing antibody titers in a non-human primate model [44].

In the last step, we tested induction of neutralizing antibodies in rabbits for the two most promising candidates, VSV-GP-gp140:G* with a mutated furin cleavage site and VSV-GP-gp140:G-linker with the native-like flexible linker. Both induced significant titers of Tier 1A neutralizing antibodies already after the prime, which were then boosted by a second immunization. The median neutralizing antibody titers increased by ~18-fold for both constructs. The third immunization did not boost the immune response further (1.1 and 1.7-fold increase of median respectively). However, no Tier 2 neutralizing antibodies were induced. In a similar study by Negril et al. using an integration-defective lentiviral vector containing 1086.C Env for immunization of NHP also only neutralizing antibodies against the clade C Tier 1 MW965.26 virus were induced, but none against the autologous 1086.C stain [45].

HIV vaccine development still faces a steep uphill battle. While HIV-infected patients are in principle capable of generating bnAbs, these antibodies are only seen in a fraction of patients and mostly at a more advanced stage of their disease [46,47]. So far none, of the HIV vaccine trials in non-human primates or humans have been capable of inducing bnAbs against a broadly neutralizing epitope on the virus. However, passive immunization strategies using bnAbs suggest that an HIV vaccine might need to induce bnAbs against several different Env epitopes in order to be efficient [48,49]. Thus, several major obstacles have been discovered that are both related to the virus [(i) exceptionally high mutation rate, (ii) low spike density, (iii) a high glycosylation level of Env and shielding of crucial epitopes for binding and induction of neutralizing antibodies [50,51]] and its host [in general absent or poor affinity of germline B cell receptors to vulnerable broadly neutralizing epitopes that were identified by epitope mapping] [47,52]. Additionally, HIV bnAbs exhibit unusual characteristics such as high frequency of somatic hypermutations, mutations in typically conserved framework regions, and abnormally long and charged complementarity determining regions [53,54,55], and may be autoreactive [56]. Recent discoveries and still ongoing research into the precise steps of the conformational changes that occur when HIV engages a target cell [57,58,59,60] will help to intelligently design trimeric Env antigens that facilitate a more efficient induction of bnAbs (e.g., arrest or more efficiently present specific conformational changes that are targets for bnAbs). In fact novel, more promising Envs have already achieved a major advance; these strategies can now induce neutralizing antibodies against Tier 2 viruses [61,62,63,64]. However, at this point, only neutralization of autologous Tier 2 viruses has been achieved. We and others are currently perusing strategies to design Envs that will afford more broadly neutralization of Tier 2 viruses. However, it also might be necessary to imitate the local environment of germinal centers that apparently permits the induction of bnAbs in HIV-infected patients but so far not in vaccines. Thus it might be necessary to combine vaccination strategies with immunomodulatory agents to break tolerance and/or imitate immunopathologic changes that are correlated with the induction of bnAbs (e.g., NK cell quantity and function) [56,65,66].

VSV-GP is a promising novel, versatile vector that expands the arsenal of potential HIV vaccine candidates: the virus can deliver HIV Env in a favorable trimeric conformation, induces robust immune responses, is easy to produce at high titers, and has a good safety profile. However, it will likely be necessary to combine different approaches to generate Envs that best present critical broadly neutralizing epitopes for the induction of bnAbs and/or facilitate the induction of humoral immune responses with strong Fc receptor engagement. In order to accomplish that, heterologous prime/boost strategies such as different viral vectors, DNA and/or protein immunizations may be necessary. Studies in non-human primate models and ultimately clinical trials will be needed to judge VSV-GP’s potential to significantly advance the development of an effective HIV vaccine.

## Supplementary Materials

The following are available online at http://www.mdpi.com/1999-4915/11/2/159/s1. Supplementary Table S1. Statistical analysis for Figure 2E, Supplementary Table S2. Statistical analysis for Figure 5, Supplementary Table S3. VSV-GP-env induces Tier 1A but no Tier 2 neutralizing antibodies upon immunization of rabbits, Supplementary Figure S1. VSV-GP particles containing HIV env show a high glycoprotein density on the surface.
