# Supplementary material for: Induction of Tier 1 HIV Neutralizing Antibodies by Envelope Trimers Incorporated into a Replication Competent Vesicular Stomatitis Virus Vector
Source: Viruses. 2019 Feb 15;11(2):159. doi: 10.3390/v11020159 (PMC6409518; doi:10.3390/v11020159)
Supplement: Supplementary file 1 [file viruses-11-00159-s001.pdf]

Supplementary material

Supplementary tables

Supplementary Table 1. Statistical analysis for Figure 2E

Time point	Kruskal-Wallis	gp120 vs. gp160*	gp160* vs. gp140:G*
**Prime**	3.5 x 10-7	0.00031	0.0145
**1st boost**	0.00024	0.00078	0.7451
**2nd boost**	6.4 x 10-7	0.0013	0.0022

Kruskal-Wallis test (P value cutoff: 0.05) on 3 groups followed by two 2-sided pairwise Wilcoxon Rank Sum tests (with Holm's adjustment for 2 comparisons): A) gp120 vs. gp160*; B) gp160* vs. gp140:G*. Significant test results are in red.

Supplementary Table 2. Statistical analysis for Figure 5

Time point	Kruskal-Wallis
**Prime**	0.68
**1st boost**	0.90
**2nd boost**	0.89

Kruskal-Wallis test on 3 groups (P value cutoff: 0.05). Since all three Kruskal-Wallis tests were non-significant no further 2-sided pairwise Wilcoxon Rank Sum tests were performed.

Supplementary Table 3. VSV-GP-env induces Tier 1A but no Tier 2 neutralizing antibodies upon immunization of rabbits.

	Bleed week	Negative Control (SVA-MLV)	Clade C (MW965.26) Tier 1A	Clade C (Ce1086_B2) Tier 2	Clade C (25710-2.43) Tier 2	Clade B (TRO.11) Tier 2	Clade C (Ce1176_A3) Tier 2
VSV-GP-gp140:G* 4 animals	pre-immune	<20	<20	<20	<20	<20	<20
	prime	<20	**21**	n.a.	n.a.	n.a.	n.a.
	1st boost	Ns	**364**	<20	<20	<20	<20
	2nd boost	<20	**839**	<20	<20	<20	<20
	pre-immune	<20	<20	<20	<20	<20	<20
	prime	<20	**52**	n.a.	n.a.	n.a.	n.a.
	1st boost	<20	**3530**	<20	<20	<20	<20
	2nd boost	<20	**2733**	<20	<20	<20	<20
	pre-immune	<20	<20	<20	<20	<20	<20
	prime	<20	**76**	n.a.	n.a.	n.a.	n.a.
	1st boost	<20	**986**	<20	<20	<20	<20
	2nd boost	<20	**1722**	<20	<20	<20	<20
	pre-immune	<20	<20	<20	<20	<20	<20
	prime	<20	**32**	n.a.	n.a.	n.a.	n.a.
	1st boost	<20	**505**	<20	<20	<20	<20
	2nd boost	<20	**413**	<20	<20	<20	<20
VSV-GP-gp140:G-linker 4 animals	pre-immune	<20	<20	<20	<20	<20	<20
	prime	<20	**78**	n.a.	n.a.	n.a.	n.a.
	1st boost	<20	**430**	<20	<20	<20	<20
	2nd boost	**23**	**416**	45	41	37	35
	pre-immune	<20	<20	<20	<20	<20	<20
	prime	<20	<20	n.a.	n.a.	n.a.	n.a.
	1st boost	<20	**557**	<20	<20	<20	<20
	2nd boost	<20	**492**	<20	<20	<20	<20
	pre-immune	<20	<20	<20	<20	<20	<20
	prime	<20	<20	n.a.	n.a.	n.a.	n.a.
	1st boost	<20	**521**	<20	<20	<20	<20
	2nd boost	<20	**585**	<20	<20	<20	<20
	pre-immune	<20	<20	<20	<20	<20	<20
	prime	<20	**36**	n.a.	n.a.	n.a.	n.a.
	1st boost	<20	**420**	<20	<20	<20	<20
	2nd boost	**26**	**1058**	<20	**26**	<20	<20
	CH01-31 (µg/mL)	>25	**2,53**	**2,66**	**0,22**	**0,13**	**0,07**

New Zealand White rabbits (n=4) were immunized intramuscularly with 2×10^{8} $TCID_{50}$ of VSV-GP-gp140:G* or VSV-GP-gp140:G-linker in weeks 0, 3 and 6. Serum samples prior to the first immunization and three weeks after each immunization were analyzed for titer of neutralizing antibodies using a TZM-bl neutralization assay. As a negative control for the neutralizing antibody assay MLV-pseudotyped viruses were used. Values in bold grey type are background signal against the MLV-pseudotyped negative control virus. Values in bold black type are considered positive bases on the criteria of signal ≥ 3x that of the same sample against the negative control virus. n.a.: not analyzed

Supplementary figures

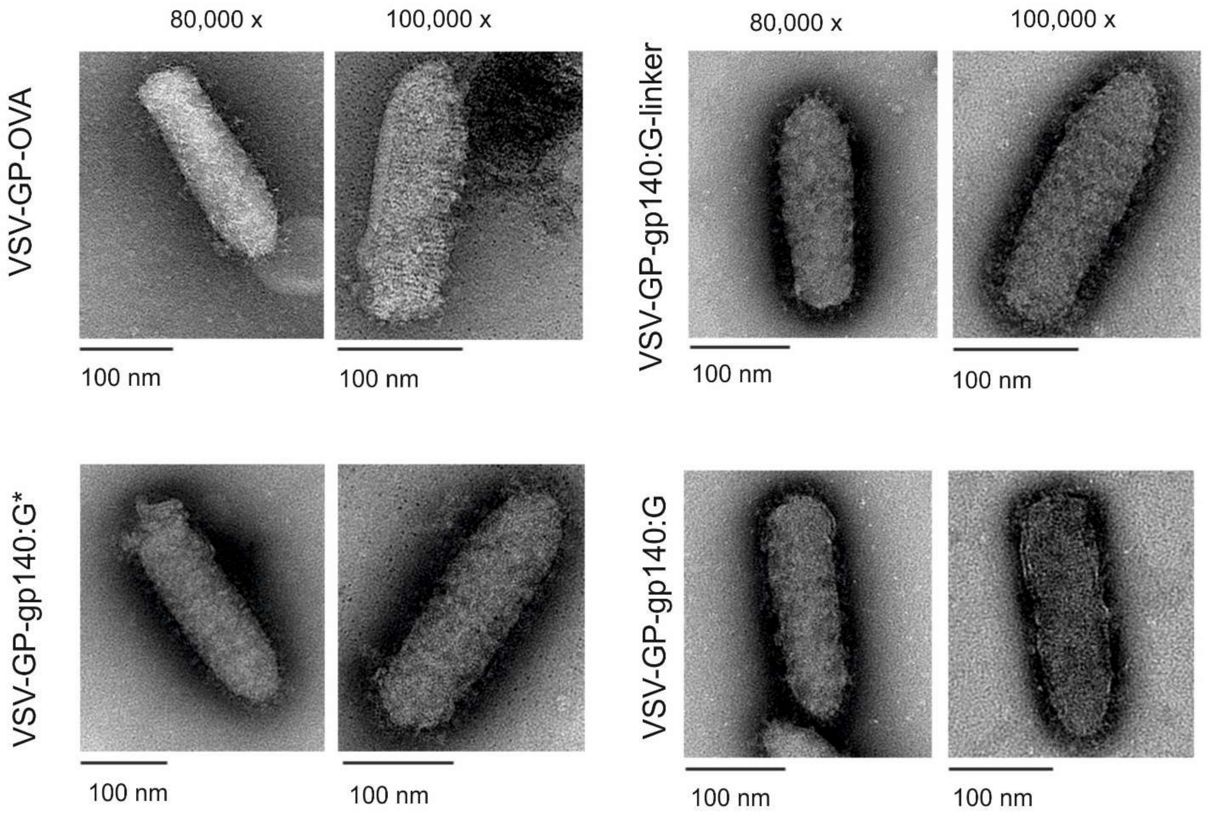

Supplementary Figure 1. VSV-GP particles containing HIV env show a high glycoprotein density on the surface. VSV-GP-gp140:G* (lower left), VSV-GP-gp140:G (lower right) and VSV-GP-gp140:G-linker (upper right) were analyzed for glycoprotein density on the surface using electron microscopy. As a negative control, VSV-GP-OVA was used (upper left). Exemplary pictures for all viruses at 80,000 and 100,000 fold magnification are shown.
